# Older African Americans’ Perspectives on Exposure to Structural Discrimination Across Contexts and the Life Course

**DOI:** 10.1093/geroni/igaa057.2407

**Published:** 2020-12-16

**Authors:** Sarah LaFave, Sarah Szanton, Roland Thorpe

**Affiliations:** Johns Hopkins University, Baltimore, Maryland, United States

## Abstract

This presentation reports on findings from the first phase of a mixed methods study aimed at developing an instrument to assess older African Americans’ exposure to structural racial discrimination. We conducted semi-structured interviews with older African Americans about their perspectives on and exposure to structural discrimination. Participants (n=20) were community-dwelling African Americans aged fifty and older in Baltimore, MD. Participants described exposure to structural discrimination that had accumulated across the life course and across the contexts of education, employment, healthcare services, criminal justice system, neighborhood factors, media and marketing of unhealthy products, environmental toxin exposures, and income, credit and wealth. In the next phase of the study, we will incorporate these findings into the development of instrument items. Developing and testing a tool to assess exposure to discrimination beyond the interpersonal level is an important step in identifying solutions to mitigate the contribute of discrimination to racial health disparities.

